# A new approach in analyzing the accident severity of pedestrian crashes using structural equation modeling

**DOI:** 10.5249/jivr.v13i1.1545

**Published:** 2021-01

**Authors:** Ali Tavakoli Kashani, Mahsa Jafari, Moslem Azizi Bondarabadi

**Affiliations:** ^*a*^ School of Civil Engineering, Iran University of Science & Technology, Tehran, Iran.; ^*b*^ Road Safety Research Center, Iran University of Science & Technology, Tehran, Iran.

**Keywords:** Pedestrian, Accident size, Structural equation modeling

## Abstract

**Background:**

According to official statistics in Iran, there were 17000 fatalities in road traffic crashes in 2018 that 25% of all crash fatalities belong to pedestrians. In most of the researches related to pedestrians’ safety, one aspect of the traffic crash (e.g. the injury or crash severity) is almost considered for the investigation. In order to perform a complete study of the crash, accident size can be utilized which involves different aspects of the crash. Accident size is described in terms of the number of fatalities and injured individuals and the number of dam-aged and involved vehicles in a crash.

**Methods:**

According to the fact that accident size has multiple indicators and it is not measured directly, traditional methodologies cannot be applied. So, in the present study the effective factors on the accident size of pedestrian crashes are investigated through structural equation modeling. For the purpose of this study, 3718 pedestrian-involved crash data occurred in Isfahan province is used for the modeling. The independent variables are weather conditions, road surface conditions, time, horizontal and vertical alignments, road type and location, driver’s gender and age, vehicle type, pedestrian’s age, gender and clothing color.

**Results:**

The results indicated that highways, the pedestrians’ invisibility, female and old-aged pedestrians, heavy vehicles, old-aged and female drivers are related to the increase of the accident size in pedestrian crashes. These results denote that the mentioned variables are associated with the higher number of injuries, fatalities, the higher number of involved and damaged vehicles in a crash.

**Conclusions:**

Present study shows the importance of considering safety improvement measures in highways, educating the people in the society about the traffic safety, the separation of pedestrian and motor vehicle traffic flow and considering the old people in policies and programs for mitigating the accident size.

## Introduction

Pedestrians are among the most vulnerable road users who often experience serious injuries in traffic crashes.^1^ The official statistics in Iran show that there were approximately a total of 17000 fatalities in traffic crashes in 2018. Among these statistics, pedestrians show noticeable susceptibility in severe crashes that approximately 25% of all crash fatalities belong to them. Because of their light and fragile bodies and low travel speeds, pedestrians involved in collisions are at a definite disadvantage relative to drivers or vehicle occupants.^2^ The investigation of influential factors on crashes enables engineers to carry out calculations in order to reduce crash severity^3^ which is an essential process in pedestrian crashes.

There are several studies addressing the safety of pedestrians in traffic crashes that in most of them the crash severity or the pedestrians’ injury severity is taken into account,^4-8,2^ which involves one aspect of the crash. However, other aspects of the crash such as the number of involved vehicles and the number of damaged vehicles can also be the representatives of the crash severity.^9^ Considering the variables related to the vehicle and injury severity simultaneously can help the researcher investigate the crash more accurately. Thus, the concept of accident size can be defined here for a thorough investigation, the indicators of which are the number of injured individuals, the number of fatalities, the number of involved vehicles and the number of damaged vehicles.^9^

Therefore, for performing a comprehensive study of the pedestrian crashes and their obvious susceptibility in the event of a crash, the objective of this study is to investigate the influential factors on the accident size of pedestrian crashes considering the factors related to road, environment, driver, pedestrian and vehicle.

**Literature review**

In previous studies, different analytical methods are employed for investigating the factors influencing the pedestrian crashes which involves Logistic regression,^4,5,10,11^ Logit^6,7,12-14^ and Probit ^15-17^ models. Prior studies have reported that drivers’ and pedestrians’ age and gender,^4,6,10,15,16,18,19^ vehicle type^4-6,10,13,14,19^ road characteristics like road type and alignments,^4,7,13,18,19^ and environmental characteristics such as time and weather conditions^7,12,16,19^ significantly affect pedestrians’ safety in road traffic crashes. 

The concept of accident size was first developed by Lee et al. (2008) that was defined using the variables of the number of injured individuals, fatalities, involved vehicles and damaged vehicles in a traffic crash. Lee et al., (2008) used 2649 accident data occurred on highways in Korea and estimated relationship among different factors and traffic accident size. The model suggested that road factors, driver factors and environment factors are strongly related to the accident size. Then, accident size was used by Hassan et al. (2013) for investigating the accident size of fatal and injury traffic crashes and the results showed that road factor was the most significant factor affecting the size of the crash followed by the driver and environment factors.^20^ In a recent study, Yuan et al. (2019) studied the accident size of fatal accidents involving trucks that the results indicated that main indicators affecting accident severity are: environmental and roadway factors such as weather and surface conditions; vehicle factors such as truck weight and body type; and driver factors such as age, driving experience, and history of crashes or convictions.^21^

According to previous studies, researchers have mainly focused on the pedestrian crashes by using different methodologies and by focusing on one feature of the crash at a time. Although there are numerous studies in the severity of pedestrian crashes, there are few studies addressing the accident size of pedestrians. So, in the present study, for investigating the crash severity more comprehensively, the relationships of human, environment, road factors and accident size are studied.

## Methodology

The goal of this study is to investigate the effects of different factors on the accident size. The accident size is considered as a representative of the crash severity which cannot be measured directly. As accident size is a linear combination of four indicators (i.e. the factors of pedestrian, vehicle, environment and road), the most proper modelling technique is the structural equation modeling (SEM). In SEM, unobservable latent variables are estimated from observed indicator variables, and the focus is on the estimation of the relations among the latent variables free of the influence of measurement errors.^22^ Observed variables are the measured variables in the data collection process and latent variables are the variables measured by connecting to the observed variables, because they cannot be directly measured. SEM has some advantages over traditional statistical methods (e.g. multiple regressions, analysis of variance, etc.) that some of them are: the ability to model multiple dependent variables simultaneously; the ability to test overall model fit, complicated and specific hypotheses, and the ability to handle difficult data (e.g., non-normal, count and categorical outcomes).^22^ For this reason, it can be said that structural equation modeling is more suitable for testing the hypothesis than other methods.^23^

**Figure 1:**
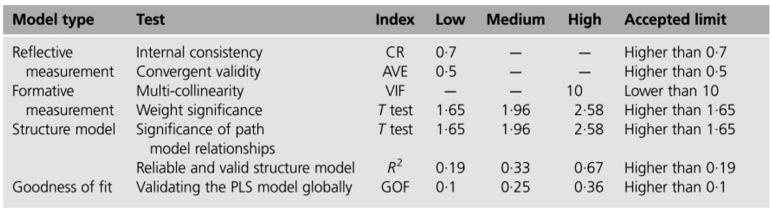
Criteria for evaluating the PLS-SEM.

There are two types of SEM: covariance-based SEM (CB-SEM) and partial least squares SEM (PLS-SEM; also called variance based SEM). CB-SEM is primarily used to confirm (or reject) theories (i.e., a set of systematic relationships between multiple variables that can be tested. The proper method can be selected with respect to the objectives of the study, and the type and the size of data used.^24^ There are some rules of thumb for choosing between CB-SEM and PLS-SEM, some of which are worth mentioning here that when the structural model is complex, sample size is small, the data are non-normally distributed or the feature of the study is exploratory the researcher should use the later one.^24^ Regardless of the approach used, an SEM is validated in three steps: the assessment of the measurement models, the assessment of the path model and the assessment of the goodness of fit of the overall model that the commonly used criteria for assessing the PLS-SEMs are provided in Figure 1.^25^

**Data description**

In the current study, the data related to the drivers, pedestrians, environmental conditions and road characteristics of the pedestrian crashes occurred on the urban roads of Isfahan province in Iran during the 2012-2016 period was used. After eliminating the incomplete cases, 3718 valid case records were obtained for the modeling. 

For the purpose of the current study, different sets of variables were extracted from the dataset. Several variables related to the environmental context, such as weather conditions, road surface conditions, and the time of occurrence were selected. Road characteristics including horizontal and vertical alignments, road type and location were derived. Driver and vehicle characteristics were considered based on the records of driver’s gender, driver’s age and vehicle type. Finally, the variables of clothing color, age and gender were considered for the pedestrians. Table 1 provides the statistical description of the measured variables. The variables of “weather conditions”, “road surface conditions”, “horizontal alignment” and “vertical alignment” did not get adequate frequency for the modeling. So, they were removed from the modeling process. 

**Table 1:** Descriptive statistics of the indicators.

Variable	Coding	Frequency	Percentage(%)
**Road characteristics**			
Road type	Two-way undivided:1	1412	38
	Two-way divided:2	1786	48
	one-way:3	520	14
Horizontal alignment	straight:1	51	1.4
	curve:2	3667	98.6
Vertical alignment	Level:1	31	0.8
	Slope:2	3687	99.2
Location	Minor road:1	520	14
	Major road:2	3121	83.9
	Highway:3	77	2.1
**The environmental characteristics**			
Weather conditions	Unclear (rainy, snowy, …):1	24	0.6
	Clear:2	3694	99.4
Surface conditions	Dry:1	3690	99.2
	Wet:2	28	0.8
Time	Day :1	2661	71.6
	Night :2	1057	28.4
**Driver and vehicle characteristics**			
Vehicle type	motorcycle:1	559	15
	auto:2	2999	80.7
	heavy vehicle:3	160	4.3
Gender	Female:1	471	12.7
	Male:2	3247	87.3
Age	Young (<31):1	1633	43.9
	middle-aged (31-55):2	1817	48.9
	old (>55):3	268	7.2
**Pedestrian characteristics**			
Clothing color	Not contrasting:1	1590	42.8
	Contrasting :2	2128	57.2
Gender	Female:1	1715	46.1
	Male:2	2003	53.9
Age	Child (3-11):1	554	14.9
	teen (12-19):2	219	5.9
	Young (20-34):3	818	22
	middle-aged (35-54):4	988	26.6
	old (>55):5	1139	30.6

## Results and Discussion

**Figure 2:**
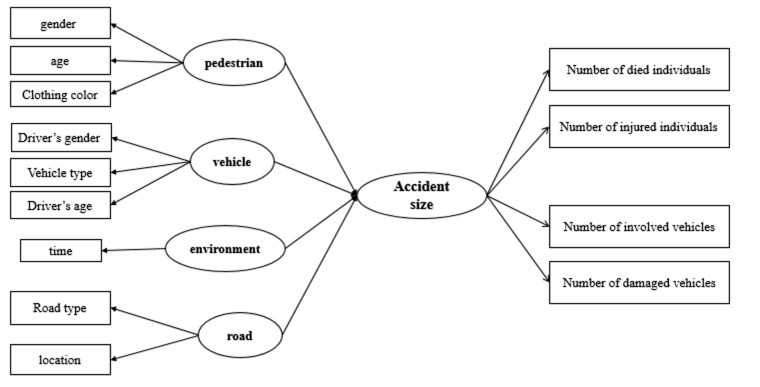
Initial structural model.

The initial model for the relationships between the considered factors was developed based on the research hypothesis: “The latent factors of vehicle, road, pedestrian and environment affect the accident size significantly”, which is illustrated in Figure 2.

The type of analysis is exploratory because there is no prior hypothesis about factors and their relationships. Consequently, the PLS-SEM approach is used in this study. There are four latent constructs: vehicle, road, pedestrian and environment to formulate the accident size. The “vehicle” factor is a construct made of the variables of vehicle type, driver’s gender and driver’s age. The “Road” construct is a combination of two variables that measures the road type and its location. “Environment” is composed of the time. Pedestrian’s gender, age and clothing color define the pedestrian factor. Finally, the accident construct (accident size) is measured by four defining variables: number of damaged vehicles, number of involved vehicles, total number of injured people and total number of fatalities. So, SEM was developed for specifying the relationships between observed and latent variables. The results of assessing the reflective measurement models are provided in Table 2.

**Table 2:** Assessment of the measurement models.

factor	AVE	Composite Reliability
Environment	1	1
Road	0.755	0.504
Vehicle	0.789	0.586
Pedestrian	0.739	0.503

According to the values of measurement models’ assessment in Table 2, all of them are compatible with the standard ranges. The accepted ranges for the indexes are mentioned in Figure 1 that CR^1^ higher than 0.7 and AVE^2^ higher than 0.5 are required.^25^ Therefore, the reliability and the validity of the measurement models are approved. Finally, for the assessment of structural model, the values of R2 and goodness of fit are 0.83 and 0.68 respectively that meet the requirements.

Table 3 shows the standardized coefficients and it is clear that all of the mentioned effects except the environment factor are statistically significant at 95% level of confidence. The significance of variables can be interpreted through the t-test of each variable that a value higher than 1.65 is accepted for the significance.^25^ As the standardized coefficients are discussed here, the effect of each latent variable on accident size can be compared. Thus, it is indicated that the most effective factors are vehicle, road, pedestrian and environment, respectively. Hence, in order to decrease the accident size of pedestrian crashes some improvement measures dealing with vehicle and road factor can be implemented. In addition, in the measurement models of road, vehicle and pedestrian the most indicative variables are location, vehicle type and pedestrian’s clothing color, respectively. The coefficients of the study illustrate that these factors are associated with the increase of accident size.

**Table 3:** Total effects.

	Path	Estimate	T Statistics
Road	>> Accident Size	0.211*	11.80
	>> Road Type	0.105	0.478
	>> Location	0.99*	24.80
Environment	>> Accident Size	0.079*	4.73
	>> Time	1	0
Pedestrian	>> Accident Size	0.162*	5.41
	>> Age	0.499*	4.57
	>> Gender	-0.709*	6.56
	>> Clothing Color	-0.87*	6.92
Vehicle	>> Accident Size	0.872*	8.383
	>> Vehicle Type	0.956*	16.69
	>> Driver’s Age	0.408*	8.25
	>> Driver’s Gender	-0.279*	12.50

* Indicates significant estimate value at a 95% confidence level

It is illustrated that in the road factor the most influential variable is road location, which shows that highways (estimate= 0.99, t-value=24.8) influence the accident size positively. This result is in agreement with previous studies.^4,15,19^ Kim et al., (2008) have shown that two-way divided roads have better geometric design, but if a pedestrian–vehicle crash happens, a pedestrian is more likely to suffer from fatal injury most likely due to higher speed on two-way divided roads. In another study, the results illustrate that high-speed urban roadways were associated with greater severe injury probability and these roads found to be more dangerous for pedestrians, especially for pedestrians crossing the roadways.^13^ Therefore, minimizing the conflict between pedestrians and vehicles in highways through the physical design can be one of the measures for reducing the accident size of pedestrian crashes.

Considering the pedestrian characteristics, the variables of clothing color, gender and age are the most effective variables, respectively. So, for controlling the accident size, educating the people and considering the old people in designing the road infrastructures (e.g. pedestrian bridges) can be some of the effective measures. The clothing color has negative influence on the accident size (estimate=-0.87, t-value=6.92), showing that having a contrasting clothing color leads to lower accident sizes. In a study conducted by Pour-Rouholamin et al. (2016), it is represented that pedestrians who wear not contrasting clothing are more vulnerable to severe injuries compared to others who wear contrasting clothing. It is believed that wearing bright or contrasting clothes makes pedestrians more visible to drivers. According to the study of Zavareh et al. (2015) , being seen on the road is a fundamental requirement for safety of all road users, particularly vulnerable road users. On the other hand, the results of present study show that old pedestrians are more vulnerable in the event of a crash which is compatible with the results of other researches.^4-7,10,12-16,18,19^ According to the study of Haleem et al. (2015), this can be mainly due to their weak physical conditions. Kemnitzer et al. (2019) have concluded that the youngest pedestrians (0–4 years old) were associated with the lowest odds of injury, whereas the oldest pedestrians (aged 65 and older) were associated with the highest odds compared to pedestrians aged 26–64. The variable of pedestrian’s gender shows that females are involved in more severe crashes other than males which is in accordance with other studies^16,27^ and this may be attributed largely to the greater fragility of females than males due to their physiological differences, in terms of height and weight, as well as differences in resistance of the body to withstand impact.^27^

Considering the vehicle and driver characteristics, it is represented that heavy vehicles are associated with higher accident sizes (estimate=0.956, t-value=16.69). The study of Kim et al. (2008) shows that with a larger mass and a longer stopping distance, sport-utility vehicles and, in particular, trucks have greater momentum, which leads to greater impacts and more serious injuries. Besides, the driver’s gender also affects the accident significantly and female drivers are related to higher accident sizes. Finally, as it is investigated in some studies,^21,28,29^ the old-aged drivers usually experience more severe crashes and this group of drivers influence the probability of accident size positively (estimate= 0.408, t-value= 8.25). It indicates that pedestrians usually experience higher accident sizes while colliding with old-aged at-fault drivers, the reason of which can be some weaknesses in the eyesight and also longer perception-reaction time of drivers.

^1^ Critical ratio

^2^ Average variance extracted

## Conclusion

Most of the prior studies in the field of pedestrian safety have focused on one feature of the accident such as injury severity or crash severity. For filling this gap, the objective of this study was to investigate the effective factors on the accident size of pedestrian’s traffic crashes. In this regard, 3718 accident data for the pedestrian crashes was used. The considered accidents occurred on urban roads of Isfahan province between 2011-2018. The results showed the characteristics related to road, driver and pedestrian influence the accident size positively. According to the results, highways and heavy vehicles are related to higher accident sizes which shows the importance of constructing safe pathways for pedestrians while crossing the high speed roads. Furthermore, older pedestrians are involved in more severe crashes indicating that this group of people should be taken into account while designing and constructing various infrastructures. Finally, it is better to inform the people with the importance of having a proper clothing in relation to the daytime. 

The findings highlight some information about the relationships between accident size and different factors influencing the traffic accident size. This study was limited to data set related to the accidents in Iran over a 5-year period. Therefore, there are important factors such as driver’s error, driving experience and pedestrian’s behavior that are not included in this study due to the lack of information and it can be considered as a possible limitation of this investigation. For the future studies, considering driver’s reactions and behaviors, land use type, accident type, the condition of the road lights can be done for improving the model.
